# Dropout from Substance Use Disorder Treatment at a Swedish Private Care Institution and Its Associated Risk Factors

**DOI:** 10.1177/29768357251332827

**Published:** 2025-04-12

**Authors:** Kent Ehliasson, Johannes Eriksson, Riccardo LoMartire

**Affiliations:** 1 Department of Work Science, Dalarna University, Falun, Sweden; 2 Stockrosen AB, Nora, Sweden; 3 Centre for Clinical Research Dalarna, Uppsala University, Sweden; 4 School of Health and Welfare, Dalarna University, Falun, Sweden

**Keywords:** substance use disorder treatment, drop out, risk factors, private care institution

## Abstract

**Background and objectives:**

The drop-out rate for inpatient treatment for substance use disorder continues to be a significant issue. To increase the knowledge about drop out in different settings, this study’s objective was to quantify the association for the previously identified risk factors of age, sex, and time in treatment at a private care institution offering substance use disorder treatment in Sweden.

**Design and methods:**

This retrospective cohort study of clinical record data included all 1334 adult clients who were discharged from substance use disorder treatment between 1 January 2014, to 30 June 2022, at one privately operated treatment institution. Drop out was defined as treatment terminated before the planned end. The association between three potential risk factors and drop out was analysed in a multivariable logistic regression model. Estimates were reported as marginal risk ratios (95% confidence intervals).

**Results:**

Of 1334 discharged clients, 34% dropped out, corresponding to 38% of females and 33% of males. Approximately 52% of clients dropped out within 30 days, 42% dropped out between 30 and 89 days, and around 15% from 90 days and onwards. In the multivariable model, both time in treatment (3.08 [2.34, 3.83] for 30 to 89 days vs 90 days and 3.55 [2.72, 4.39] for <30 days vs ⩾90 days) and age (1.19 [1.14, 1.23] for one decade) showed a strong inverse association with drop out. The results did not support an association between sex and drop out (1.05 [0.89, 1.22]).

**Conclusion:**

The risk for drop out is higher earlier in the treatment and for younger clients, so to reduce the drop out at private institutional care it is important to implement extensive interventions early in the treatment programme to increase the motivation for clients, particularly younger ones, to remain in treatment.

## Introduction

Substance misuse constitutes a significant difficulty for the individual, their kin, and society at large.^1-3^ In order to aid individuals grappling with substance misuse, diverse forms of treatment programmes have been developed, systematically aimed at aiding the individual in diminishing their intake, managing the risk of relapse, achieving eventual drug abstinence, and enhancing their functional capacity to lead an ordinary existence.^4,5^ The arena of substance misuse care, along with its many treatment programmes, diverges significantly concerning focus, scope, and content, generally encompassing facets of pharmacological, psychological, and social interventions. The duration of treatment may span from several months to numerous years.^4-6^ However, the duration, content, and post-treatment monitoring exhibit notable heterogeneity.^7,8^

A crucial determinant for achieving drug abstinence resides in successfully undergoing and completing a programme of treatment, a facet interconnected with numerous dimensions such as reduced criminality, higher employment rates, diminished relapses into substance misuse, and the like.^4,9^ Simultaneously, it is evident that drop outs are prevalent in substance misuse care, with attrition rates ranging between 32% and 68% among clients.^10,11^ Among detoxification patients, treatment discontinuation was observed in 22% to 43% of cases.^12,13^ In the context of inpatient care, approximately 17% to 88% of patients dropped out ^14-18^ while within outpatient settings, roughly 23% to 50% discontinued their care.^19,20^ In summation, it could be stated that drop-out rate in substance use disorder treatment varies considerably between studies but is a prevailing phenomenon.^5^

Among the risk factors that can be associated with drop out, this study was focused on clients’ age, sex, and their time in treatment since these aspects are more common, consistent, and could be more manageable to apply to and replicate in new settings compared to more complex risk factors such as client’s diagnosis, treatment methods, and organisational settings. Regarding clients’ age and dropping out, studies clearly support the notion that age correlates negatively with treatment drop out.^5,16,21^ However, several studies did not find age differences between those who dropped out and those who completed the treatment, and other studies have identified that older alcoholics are more prone to relapse than younger ones.^18,22^ Concerning sex and drop out from treatment programmes, research shows diverse outcomes; some indicate a predominance of women among premature discharges,^23^ while others suggest a slight overrepresentation of men.^24^

In substance use disorder treatment, it has been shown that individuals with substance dependence exhibit a lesser propensity to attain a positive treatment outcome during the initial phase of their intervention, and a successful outcome progressively increases if the client’s duration of stay in treatment exceeds 3 months.^25-28^ Given that drop out occurs for over 50% of clients within the first month of treatment,^29,30^ clients do not have sufficient time to recover from substance misuse, receive adequate exposure to treatment, establish a functional therapeutic alliance with staff, create favourable conditions, and instil hope for achieving drug abstinence.^29,31^ It can be observed that factors enhancing the possibility of clients remaining in treatment encompass swift initial response and individual attention, smaller groups, decentralised units, more conveniently located facilities with higher clinical staff-to-patient ratios, and greater per capita expenditure.^29^

In the Swedish context, a study has shown that 59% of clients from year 2001 to 2009, assigned to compulsory care – annually 798 people, 478 male (60%) and 320 female (40%) – dropped out and that younger individuals were significantly more likely to drop out.^32,33^ However, the majority of individuals who received substance use disorder treatment in institutional care settings did so voluntarily and comprised around 6348 people annually: 4218 (66%) male and 2130 (34%) female.^34^ Even though most of the clients received voluntary treatment in institutional care there is a lack of knowledge in a Swedish context regarding dropout and risk factors age, sex and time in treatment, which has been showed in previous research, within a private care institution for drug treatment. There are reasons to believe that these risk factors are similar in the Swedish context, based on earlier studies in different settings, but since the care system in Sweden varies from other countries, there could be reasons to believe there are differences.

Therefore, this study investigated if the risk factors shown in earlier research was similar or different in the voluntary inpatient treatment care settings in Sweden. Hence, the aim of this study was to extend the current knowledge and to quantify the association between previously identified risk factors such as age, sex, and time in treatment and drop out in a Swedish context. By replicating international research in Sweden, this study can increase the knowledge of whether clients with substance use disorder have similar risk factors for drop out in voluntary institutional care settings in different contexts.

## Material and Methods

### Design

This was a retrospective cohort study of clinical record data involving all 1334 adult clients who were discharged from substance use disorder treatment provided by one privately operated treatment institution, containing six units, during the period of 1 January 2014 to 30 June 2022. This implied that a client admitted before 31 December 2013 was included in the study if they were discharged before 30 June 2022. Clients admitted between 1 January 2014, and 30 June 2022, who had not been discharged, were therefore not included in the study.

This study was approved by the Swedish Ethical Review Authority 2023-05-03 (ethical approval number 2023-02276-01-404309), acting on *Act (2003:460) concerning the ethical review of research involving humans*,^35^ and performed in accordance with the ethical principles for research involving human subjects according to the updated Declaration of Helsinki. Since this was a retrospective study without intervention and all information retrieved was aggregated and fully confidential, ensuring that no individual client could be linked to the study and that the research involves a negligible risk of harm and minimal discomfort for the research participant, the authority waived the need for informed consent from the participants.

### Setting

In Sweden, both health care organisations and social services are responsible for addiction treatment. Health care mainly provides medical treatment and rehabilitation for individuals with substance misuse, while social services offer support such as housing, outpatient care, and various social interventions.

In Sweden, the initiation of voluntary and mandatory care is basically initiated by the municipality’s social services board. The compulsory care is limited to 6 months and the voluntary care has no time limits. There are also common, at the end of the compulsory care time, that clients are placed at a voluntary care institution. The voluntary care can be provided by private or public institutions and the compulsory care is provided by the state. The various private entities with authorised licenses to provide voluntary care and treatment, are referred to as ‘homes for care or living’ (HVB). A HVB offers treatment or is oriented towards caregiving, support, or guidance and encompasses a range of diverse institutions which vary in operational structure, target groups, methodologies, different treatment programmes etcetera. There were around 200 HVB licensed to provide addiction treatment for individuals from 18 years and older in 2015.^36^ Hence, this study examines clients within the context of substance use disorder treatment in Sweden, with a specific focus on a private provider offering voluntary care for adults with substance use disorder.

#### The Private Care Institution

The institution that was chosen provided substance use disorder treatment for adults, had a large number of beds with a significant number of clients passing through during the study period, had an accessible clinical record database over a time that created a more substantial base for data analysis, had accessible treatment methods for the clients, had a willingness to participate in the research, and was available to participate at the given time.

The provider operated 157 care beds distributed across six care units, varying in size from 47 beds to seven. Generally, the clients exhibited a complex pattern of substance misuse, often involving multiple types of drugs. The study includes all clients from all care units, encompassing various forms of substance misuse, comorbidity, as well as both voluntary and compulsory care. The treatment primarily consisted of cognitive behavioural therapy (CBT) and a 12-step programme. All care was tailored to the individual client’s circumstances, which meant that the care efforts could vary significantly between clients.

Unit 1 had 47 beds for men and women and treatments included CBT in the form of relapse prevention, motivational interviewing (MI), and 12-step treatment and was offered both in groups and individually. Unit 2 had 12 places for women and treatment was based on 12-step treatment and took place both in groups and individually. Unit 3 was a mixed-sex unit with 34 places and treatment was offered through CBT both in groups and individually. Unit 4 had 21 beds for men and treatment was based on CBT and 12-step treatment both in groups and individually. Unit 5 was a detoxification unit with seven beds for men and women, and some individual treatment took place in the form MI. Unit 6 had 28 places, was a mixed-sex unit, and was a special psychiatric home for mentally disabled people with substance misuse. Active treatment, individually with CBT, was only given to those clients who were motivated.

### Data Source

Upon admission to the healthcare provider, clients were admitted and recorded in a shared electronic clinical record system across all units. Information such as sex, age, legal basis for placement, municipality of origin, contact details of relatives, medical history, and substance misuse history, were registered here. However, factors like race, social class, and ethnicity were not recorded by the organisation.

The study’s data was derived from the healthcare provider’s electronic clinical record system, which was supplied by an external vendor. A completely de-identified dataset containing only the information relevant to the study’s objectives was created and transferred to the statistical programme. Drop out was defined as patients who did not complete their planned treatment programme. By this concept, we refer to all reasons for treatment noncompletion, including those stemming from the client, the current treatment facility, or the social service.

### Data Analysis

The outcome was treatment dropout, defined as discontinuation of treatment before completion, as recorded in the patient’s journal by healthcare personnel. Explanatory variables included time in treatment, measured from entry to exit at the healthcare unit, attained age, and sex, all documented by healthcare personnel.

Descriptive statistics were presented as mean (standard deviation; min-max) and frequency (percent) for continuous and categorical variables, respectively. Multivariable logistic regression was used to analyse the association between three previously identified risk factors and drop out: sex (as a categorical variable with the levels male and female), attained age (as a continuous variable, assuming a linear relationship), and time in treatment (as a categorical variable with the levels: <30 days, 30 to 89 days, and ⩾90 days). The linearity assumption was assessed visually in a quantile-quantile plot based on the model’s randomized quantile residuals.^37^ Potential dependencies within clinics were managed by robust (Huber-White) standard errors.^38^ Finally, estimates were converted to risks by marginal standardisation and reported as marginal risk ratios with 95% confidence intervals by the delta method.^39^ All analyses were conducted in the statistical software R v4.4.1 using the libraries sandwich v3.1-1, statmod v1.5.0, and marginaleffects v0.22.0.

### Results

The study consisted of 1334 clients of whom 1006 (75%) were male and 328 (25%) were female. Of all the clients, 874 (66%) completed their treatment and 460 dropped out (34%).

#### Time in Treatment

Table 1 shows that the mean time in treatment was 127 days (SD = 420), minimum 1 day and maximum 8813 days. For the clients that completed their treatment, the mean time was 157 days (SD = 491), minimum 1 day and maximum 8813 days. The clients that dropped out were in treatment for a mean time of 70 days (SD = 219): minimum 1 day and maximum 3679 days. Mean difference between the groups was 87 days. Approximately 216 (52%) of 419 clients dropped out within 30 days, 175 (42%) of 418 dropped out between 30 and 89 days, around 14% from 90 to 179 days, 15% from 180 to 365 days, and 17% for more than 365 days. Of all 1334 discharged clients, 460 dropped out and, among them, 391 (85%) did it within the first 90 days, which clearly highlights that it was more common to drop out early in the treatment than later and the risk of drop out was lower with longer placement durations. Our result support that time in treatment was inversely associated with the risk for drop out. For time in treatment, the drop-out risk was 3.55 (95% CI: 2.72, 4.39) times higher within the first 30 days and 3.08 (95% CI: 2.34, 3.83) times higher for days 30 to 89 when compared to prolonged treatment for more than 90 days, adjusted for age and sex.

**Table 1.** Sample characteristics.

	Complete sample (n = 1334)	Completed treatment (n = 874)	Dropped out (n = 460)
*Unit*			
1	669	431 (64%)	238 (36%)
2	57	23 (40%)	34 (60%)
3	163	109 (67%)	54 (33%)
4	192	111 (58%)	81 (42%)
5	99	84 (85%)	15 (15%)
6	154	116 (75%)	38 (25%)
*Days in treatment mean* (SD), min/max	127 (420), 1/8813	157 (491), 1/8813	70 (219), 1/3679
<30	419	203 (48%)	216 (52%)
30-89	418	243 (58%)	175 (42%)
⩾90	497	428 (86%)	69 (14%)
*Age in years*, mean (SD), min/max	43 (15), 18/89	45 (16), 18/89	39 (14), 18/77
<30	333 (25%)	185 (56%)	148 (44%)
31-39	310 (23%)	177 (57%)	133 (43%)
40-49	210 (16%)	140 (67%)	70 (33%)
50-59	247 (19%)	183 (74%)	64 (26%)
⩾60	234 (18%)	189 (81%)	45 (19%)
*Sex*			
Male	1006	670 (67%)	336 (33%)
Female	328	204 (62%)	124 (38%)

#### Age

In Table 1, it shows that the clients’ mean age was 43 years (SD = 15), minimum 18 years and maximum 89 years, and 643 (48%) of all clients were younger than 40 years. For the clients that fulfilled their treatment, the mean age was 45 (SD = 16), minimum 18 and maximum 89 years. The mean age for the clients that dropped out was 39 years (SD = 14), minimum 18 and maximum 77 years. Mean difference between the groups was 6 years. Clients that dropped out from treatment were considerably more likely to be younger than 40 years: the drop-out rate for younger than 30 years was 44%, between 30 and 39 years: 43%, between 40 and 49 years: 33%, between 50 and 59 years: 26%, and 60 years and older: 19%. Our results support that age was inversely associated with the risk for drop out. A decade’s decrease in age corresponded to 1.19 (95% CI: 1.14, 1.23) times the risk increase, adjusted for time in treatment and sex.

#### Sex

Among females, 124 (38%) and among males, 336 (33%) dropped out (Table 1). Women were slightly overrepresented in relation to men in dropping out during their treatment programme. Our study found no support for an association between sex and drop out (1.05 [0.89, 1.22]), adjusted for time in treatment and age. The logistic regression model together with the visual assessment of the model linearity assumption are presented in the supplementary materials (Supplemental Table S1, Figure S1).

## Discussion

This study found that out of totally 1334 clients, 66% successfully completed the treatment while 34% dropped out. Notably, the risk of drop out was more than three times higher within the first 90 days compared to later. Younger age was also associated with an increased dropout risk, with a nearly 20% relative risk increase for one decade decrease in age. Meanwhile, no support was found for an association between sex and drop out.

Approximately half of the clients dropped out within the first 30 days of treatment, aligning with previous research showing that over 50% of dropouts occur within the first month.^29,30^ Moreover, 85% of all dropouts in this study occurred within the first 90 days, after which the dropout rate drastically decreased to around 15%. These findings are consistent with prior studies indicating that treatment success increases markedly when a client’s stay exceeds 3 months.^25-28^ Our results thus emphasize the importance of preventing early dropout within the first months of treatment. Possible explanations for the early dropout include the need to return to work, provide for family, or avoid the risk of losing housing.^29,31^ Additionally, some clients may not have sufficient time to recover from substance misuse, manage intense drug cravings, or receive adequate exposure to treatment.^29,31^ Challenges in establishing a strong therapeutic alliance with staff, creating supportive conditions, and fostering hope for achieving drug abstinence may also contribute to early discontinuation.^29,31^ This underlines the importance of future investigations into reasons for the clients to stay in treatment and other factors like treatment facility settings, and group size, which can prevent clients from dropping out from the treatment programme.^29,31^

Our findings aligns with some previous research showing that increasing age is negatively associated with treatment discontinuation.^5,16,21^ Conversely, other studies have both found no association of age and that older individuals with alcohol use disorder are more prone to relapse than younger ones.^18,22^ To better understand these inconsistencies, further research should explore additional factors that may influence dropout rates. Factors could include whether younger clients experience a heavier burden of psychiatric issues, undergo shorter detoxification, use different substances, or have not yet faced the full consequences of long-term drug use.

Even though no support was found for an association between sex and drop out, this study indicates a slight overrepresentation of female, 38%, in relation to male, 33%, when it comes to dropouts. Previous research points in different directions: some suggest an overrepresentation of women among dropouts^23^ while others indicate a slight overrepresentation of men.^24^ Whether the females’ drop out translates to a greater risk of treatment drop out is uncertain, but it could be further investigated with a focus on differences between females and males regarding psychological difficulties, parenthood, social situation, and response to different treatment programmes.

The dropout rate in our study is consistent with the lower range reported in previous research for inpatient care, which showed that approximately 17% to 88% of inpatients drop out.^14-18^ The fact that our study’s results fall within the lower range for inpatient treatment is evident, although explaining this phenomenon is not straightforward. Even though the drop out rate in is in the lower range in this study, and compared to the drop-out rate in compulsory care (58%) in Sweden,^32^ the voluntary care was significantly lower (34%); yet the considerable amount still indicates the importance of underlining the necessity for measures to reduce drop out. Thus, one possible research direction in the future is to further investigate these risk factors among other providers (private and public) for institutional voluntary misuse treatment in the Swedish context or in other settings. Another research direction could be to examine post-dropout outcomes by following clients over an extended period through registry studies, comparing those who dropped out with those who completed treatment.

A significant strength of this study is the relatively large sample size and the ability to track clients from enrolment to discharge. Additionally, the clinical records provided relevant information on key characteristics such as dropout, age, gender, and time in treatment. The inclusion of all clients within a specific timeframe minimizes selection bias, while the adequacy of clinical record data reduces potential information bias. However, the use of clinical records also presents limitations, as the accuracy of reported information cannot be guaranteed, and some relevant details may not have been registered.^40^ Furthermore, it remains unclear to what extent dropout is influenced by facility characteristics, treatment duration, or background variables such as psychiatric conditions, substance use patterns, and legal placements. Another important limitation is that we cannot be certain the groups compared in our study are equivalent in all characteristics, and unmeasured characteristics could provide an alternative explanation for our findings. Finally, no a priori sample size calculation was performed; however, the sample was sufficiently large to ensure reliable parameter estimation. Despite these limitations, our results suggest that the Swedish context aligns with previous research on individuals with drug misuse in various settings. This indicates that the findings may be generalizable to individuals with substance use disorders more broadly and, to some extent, to those receiving voluntary treatment in private institutions, particularly in Sweden. However, generalizability to other institutions may be limited due to differences in factors such as institutional size, staff-to-patient ratios, treatment focus, and client diagnoses.

## Conclusions

This retrospective cohort study, based on clinical record data from 1334 adult clients discharged from a private institution investigated the associated between several previously identified risk factors for dropout. Our findings demonstrate that dropout risk is highest early in treatment and among younger clients. To mitigate this risk, it is crucial to implement targeted interventions early in the treatment program, particularly for younger clients, to enhance motivation and encourage retention in care.

## Supplemental Material

sj-docx-1-sat-10.1177_29768357251332827 – Supplemental material for Dropout from Substance Use Disorder Treatment at a Swedish Private Care Institution and Its Associated Risk Factors Supplemental material, sj-docx-1-sat-10.1177_29768357251332827 for Dropout from Substance Use Disorder Treatment at a Swedish Private Care Institution and Its Associated Risk Factors by Kent Ehliasson, Johannes Eriksson and Riccardo LoMartire in Substance Use: Research and Treatment

## Statements and Declarations

**Ethical Considerations:** This study was approved by the Swedish Ethical Review Authority 2023-05-03 (ethical approval number 2023-02276-01-404309), acting on *The Swedish Act (2003:460) concerning the ethical review of research involving humans*.

**Consent for Publication:** Written informed consent for publication was provided by all the participants.

**Data Availability:** Data can be made available upon reasonable request.

## References

[1] UN. World Drug Report 2016. United Nations Office on Drugs and Crime; 2016.

[bibr2-29768357251332827] Degenhardt LP Hall WP. Extent of illicit drug use and dependence, and their contribution to the global burden of disease. Lancet (Brit ed). 2012;379(9810):55-70. 10.1016/S0140-6736(11)61138-0 22225671

[3] Whiteford HAP Degenhardt LP Rehm JP , et al. Global burden of disease attributable to mental and substance use disorders: findings from the Global Burden of Disease Study 2010. Lancet (Brit ed). 2013;382(9904):1575-1586. 10.1016/S0140-6736(13)61611-6 23993280

[4] Kleber HD Weiss RD Anton RF Jr , et al. Treatment of patients with substance use disorders. Am J Psychiatry. 2007;164(4 Suppl.):5-123. 17569411

[bibr5-29768357251332827] Brorson HH Ajo Arnevik E Rand-Hendriksen K Duckert F. Drop-out from addiction treatment: a systematic review of risk factors. Clin Psychol Rev. 2013;33(8):1010-1024. 24029221 10.1016/j.cpr.2013.07.007

[6] Landry MJ. Overview of Addiction Treatment Effectiveness. US Department of Health and Human Services, Public Health Service, Substance Abuse and Mental Health Services Administration, Office of Applied Studies.; 1996.

[7] Porter L Arif A Curran WJ. The Law and the Treatment of Drug-and Alcohol-Dependent Persons: A Comparative Study of Existing Legislation. World Health Organization; 1986. 10.1016/0160-2527(86)90036-1 3957525

[8] WHO. Drug and Alcohol Dependence Policies, Legislation and Programmes for Treatment and Rehabilitation. World Health Organization; 1999.

[9] Dalsbø TK Hammerstrøm KT Vist GE , et al. Psychosocial interventions for retention in drug abuse treatment. Cochr Datab Syst Rev. 2010;(1):CD008220

[10] Lin HC Chen KY Wang PW , et al. Predictors for dropping-out from methadone maintenance therapy programs among heroin users in southern Taiwan. Subst Use Misuse. 2013;48(1-2):181-191. 23368704 10.3109/10826084.2012.749411

[11] Smyth BP Fagan J Kernan K. Outcome of heroin-dependent adolescents presenting for opiate substitution treatment. J Subst Abuse Treat. 2012;42(1):35-44. 21940134 10.1016/j.jsat.2011.07.007

[12] Gilchrist G Langohr K Fonseca F Muga R Torrens M. Factors associated with discharge against medical advice from an alcohol and drug inpatient detoxifcation unit in Barcelona between 1993 and 2006. Heroin Addict Relat Clin Probl. 2012;14(1):35-44.

[13] Specka M Buchholz A Kuhlmann T Rist F Scherbaum N. Prediction of the outcome of inpatient opiate detoxification treatment: results from a multicenter study. Eur Addict Res. 2011;17(4):178-184. 21494045 10.1159/000324873

[14] Deane FP Wootton DJ Hsu C-I Kelly PJ. Predicting dropout in the first 3 months of 12-step residential drug and alcohol treatment in an Australian sample. J Stud Alcohol Drug. 2012;73(2):216-225. 10.15288/jsad.2012.73.216 22333329

[bibr15-29768357251332827] Samuel DB LaPaglia DM Maccarelli LM Moore BA Ball SA. Personality disorders and retention in a therapeutic community for substance dependence: personality disorders predicting retention. Am J Addict. 2011;20(6):555-562. 21999502 10.1111/j.1521-0391.2011.00174.x PMC3856923

[bibr16-29768357251332827] Saarnio P Knuuttila V. A study of risk factors in dropping out from inpatient treatment of substance abuse. J Subst Use. 2003;8(1):33-38.

[bibr17-29768357251332827] Amodeo M Chassler D Oettinger C Labiosa W Lundgren LM. Client retention in residential drug treatment for Latinos. Eval Progr Plan. 2008;31(1):102-112. 10.1016/j.evalprogplan.2007.05.008 18222144

[18] Darke S Campbell G Popple G. Retention, early dropout and treatment completion among therapeutic community admissions. Drug Alcohol Rev. 2012;31(1):64-71. 21426420 10.1111/j.1465-3362.2011.00298.x

[19] McHugh RK Murray HW Hearon BA , et al. Predictors of dropout from psychosocial treatment in opioid-dependent outpatients. Am J Addict. 2013;22(1):18-22. 23398222 10.1111/j.1521-0391.2013.00317.x PMC3651588

[20] Santonja-Gómez FJ Sánchez-Hervás E Secades-Villa R Zacarés-Romaguera F García-Rodríguez O García-Fernández G. Pretreatment characteristics as predictors of retention in cocaine-dependent outpatients. Addict Disord Their Treat. 2010;9(2):93-98.

[21] McKellar J Kelly J Harris A Moos R. Pretreatment and during treatment risk factors for dropout among patients with substance use disorders. Addict Behav. 2006;31(3):450-460. 15979244 10.1016/j.addbeh.2005.05.024

[22] Yates WR Booth BM Reed DA Brown K Masterson BJ. Descriptive and predictive validity of a high-risk alcoholism relapse model. J Stud Alcohol Drug. 1993;54(6):645. 10.15288/jsa.1993.54.645 8271799

[23] Andersson HW Steinsbekk A Walderhaug E Otterholt E Nordfjærn T. Predictors of dropout from inpatient substance use treatment: a prospective cohort study. Subst Abuse Res Treat. 2018;12:1-10. 10.1177/1178221818760551 PMC5843095 29531472

[24] Lappan SN Brown AW Hendricks PS. Dropout rates of in-person psychosocial substance use disorder treatments: a systematic review and meta-analysis. Addict (Abingd, Eng). 2020;115(2):201-217. 10.1111/add.14793 31454123

[25] Simpson DD. The relation of time spent in drug abuse treatment to posttreatment outcome. Am J Psychiatry. 1979;136(11):1449. 10.1176/ajp.136.11.1449 495799

[bibr26-29768357251332827] Simpson DD. Treatment for drug abuse: follow-up outcomes and length of time spent. Archiv Gen Psychiatry. 1981;38(8):875-880. 10.1001/archpsyc.1981.01780330033003 7259424

[bibr27-29768357251332827] Eaton L. Numbers starting treatment for drug misuse increase by 20% over two years. Br Med J. 2004;329(7474):1066. 10.1136/bmj.329.7474.1066-e PMC526155 15528613

[28] Hawkins EJ Baer JS Kivlahan DR. Concurrent monitoring of psychological distress and satisfaction measures as predictors of addiction treatment retention. J Subst Abuse Treat. 2008;35(2):207-216. 18082998 10.1016/j.jsat.2007.10.001

[29] Stark MJ. Dropping out of substance abuse treatment: a clinically oriented review. Clin Psychol Rev. 1992;12(1):93-116.

[30] Basu D Ghosh A Sarkar S Patra B Subodh B Mattoo S. Initial treatment dropout in patients with substance use disorders attending a tertiary care de-addiction centre in north India. Ind J Med Res (New Delhi, Ind 1994). 2017;146(8):77-84. 10.4103/ijmr.IJMR_1309_15 PMC5890600 29578199

[31] Sandström S. Missbruk, trauma och samsjuklighet: bemöta och behandla. Gothia fortbildning; 2019.

[32] Padyab M Grahn R Lundgren L. Drop-out from the Swedish addiction compulsory care system. Eval Progr Plan. 2015;49:178-184. 10.1016/j.evalprogplan.2014.12.016 25563530

[33] SiS. SiS i korthet 2023. En samling statistiska uppgifter om SiS [SiS in Brief 2023. A Collection of Statistical Data on SiS]. The National Board of Institutional Care; 2023.

[34] Socialstyrelsen. Statistik om vuxna personer med missbruk och beroende [Statistics on Adults with Substance Abuse Addiction]. The National Board of Health and Welfare; 2023.

[35] SFS. SFS. 2003:460. Lag om etikprövning av forskning som avser människor [Swedish Code of Statutes 2003:460. Act Concerning the Ethical Review of Research Involving Humans]. 2003.

[36] Socialstyrelsen. Att stärka kvaliteten i hem för vård eller boende (HVB) för personer med missbruks- och beroendeproblem [To Strengthen the Quality of Homes for Care or Living (HVB) for People with Substance Abuse and Addiction Problems]. The National Board of Health and Welfare; 2015.

[37] Dunn PK Smyth GK. Randomized quantile residuals. J Comput Graph Stat. 1996;5:236-244.

[38] Zeileis A. Object-oriented computation of sandwich estimators. J Stat Softw. 2006;16(9):1-16.

[39] Muller CJ Maclehose RF. Estimating predicted probabilities from logistic regression: different methods correspond to different target populations. Int J Epidemiol. 2014;43(3):962-970. 24603316 10.1093/ije/dyu029 PMC4052139

[40] Callen JL Alderton M McIntosh J. Evaluation of electronic discharge summaries: a comparison of documentation in electronic and handwritten discharge summaries. Int J Med Inform (Shan, Ireland). 2008;77(9):613-620. 10.1016/j.ijmedinf.2007.12.002 18294904

