# Supplementary material for: Dropout from Substance Use Disorder Treatment at a Swedish Private Care Institution and Its Associated Risk Factors
Source: Subst Use. 2025 Apr 12;19:29768357251332827. doi: 10.1177/29768357251332827 (PMC12033491; doi:10.1177/29768357251332827)
Supplement: sj-docx-1-sat-10.1177_29768357251332827 – Supplemental material for Dropout from Substance Use Disorder Treatment at a Swedish Private Care Institution and Its Associated Risk Factors [file sj-docx-1-sat-10.1177_29768357251332827.docx]

**Supplementary materials**

**Table S1.** Multivariable logistic regression model.

|  | Log-odds estimate | Robust standard error | Z value | P-value |
| --- | --- | --- | --- | --- |
| Intercept | -0.496 | 0.212 | -2.339 | 0.019 |
| Sex |  |  |  |  |
| Male | *Ref* | - | - | - |
| Female | 0.090 | 0.148 | 0.613 | 0.540 |
| Age | -0.033 | 0.004 | -7.562 | <0.001 |
| Days in treatment |  |  |  |  |
| > 90 days | *Ref* | - | - | - |
| 30-89 days | 1.893 | 0.166 | 11.374 | <0.001 |
| < 30 days | 1.612 | 0.170 | 9.508 | <0.001 |


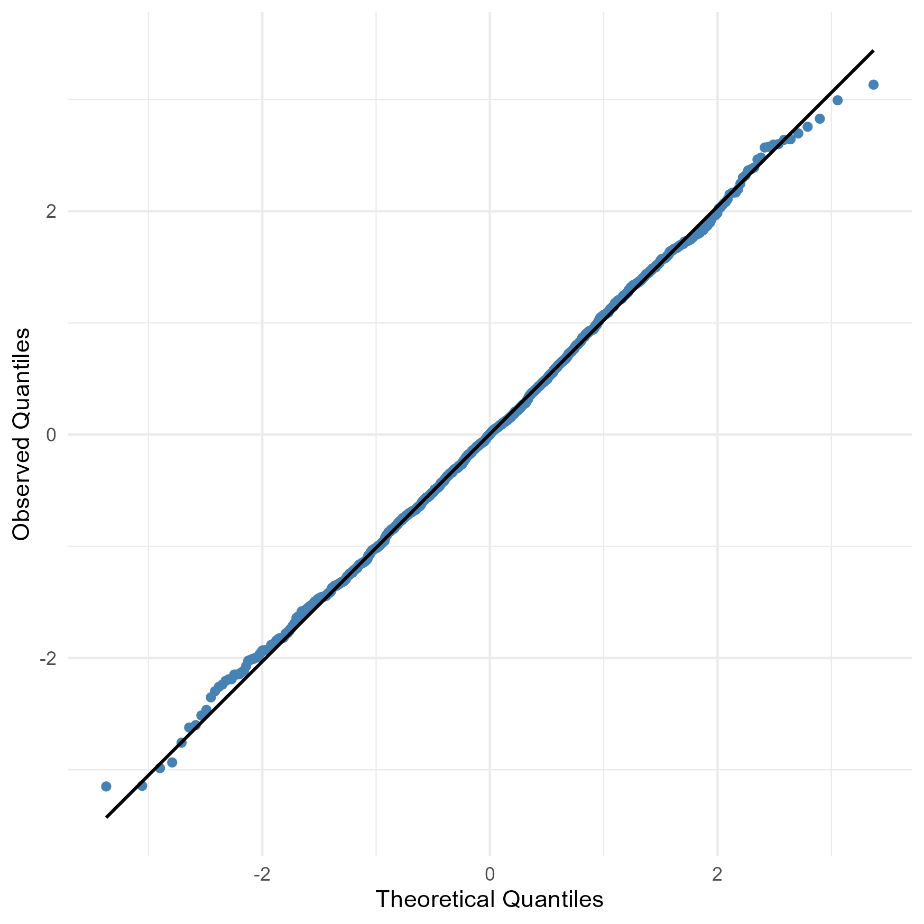


**Figure S1.** Quantile-quantile plot of the logistic regression model’s randomized quantile residuals. The visual assessment supports that the residuals are normally distributed and that the linearity assumption is met.
